# Supplementary material for: Pixel super-resolved fluorescence lifetime imaging using deep neural networks
Source: Photonix. 2026 Aug 14;7(1):56. doi: 10.1186/s43074-026-00277-9 (PMC13476302; doi:10.1186/s43074-026-00277-9)
Supplement: Supplementary file 1 [file 43074_2026_277_MOESM1_ESM.pdf]

Supplementary Information for

Pixel Super-Resolved Fluorescence Lifetime Imaging Using Deep Neural Networks

Paloma Casteleiro Costa[1,2], Parnian Ghapandar Kashani[1], Xuhui Liu[3] , Alexander Chen[1] , Ary Portes[1], Julien Bec[3], Laura Marcu[3], and Aydogan Ozcan[1,2,4,*]

[1]Electrical and Computer Engineering Department, University of California, Los Angeles, CA, 90095, USA
[2]Bioengineering Department, University of California, Los Angeles, CA, 90095, USA
[3]Department of Biomedical Engineering, University of California, Davis, California 95616, USA
[4]California NanoSystems Institute (CNSI), University of California, Los Angeles, CA, 90095, USA
[*]Correspondence to: ozcan@ucla.edu

Supplementary Note. Brownian Bridge Diffusion Process

We trained a conditional Brownian bridge diffusion model (BBDM) that translates from the low-resolution FLIM domain $I^{input} \in \mathbb{R}^{\frac{H}{k} \times \frac{W}{k} \times 6}$ to the corresponding super-resolution domain $x_0 = I^{target} \in \mathbb{R}^{H \times W \times 6}$, where k is the super-resolution factor.

The BBDM is characterized by a pair of stochastic processes: a forward diffusion process and a corresponding reverse process[1]. The forward Brownian Bridge diffusion process with total of T sampling steps is defined as

$$q(x_t \mid x_0, x_T = y) = \mathcal{N}\big((1 - m_t)x_0 + m_t y,\ \delta_t I\big) \tag{1}$$

where

$$m_t = \frac{t}{T},\ \ \delta_t = \frac{2t(T-t)}{T^2} \tag{2}$$

Given the transition probability $q(x_t \mid x_0, x_T = y)$, the intermediate states can be written as

$$x_t = x_0 + m_t(y - x_0) + \sqrt{\delta_t}\varepsilon_t \tag{3}$$

$$x_{t-1} = x_0 + m_{t-1}(y - x_0) + \sqrt{\delta_{t-1}}\varepsilon_{t-1} \tag{4}$$

where

$$\varepsilon_t, \varepsilon_{t-1} \sim \mathcal{N}(0, I) \tag{5}$$

By eliminating x_0 from Eqs. (3) and (4), the transition probability $q(x_t \mid x_{t-1}, y)$ can be derived as

$$q(x_t \mid x_{t-1}, y) = \mathcal{N}\left(x_t; \frac{1 - m_t}{1 - m_{t-1}} x_{t-1} + \left[m_t - \frac{1 - m_t}{1 - m_{t-1}} m_{t-1}\right] y, \delta_{t|t-1} I\right) \tag{6}$$

where

$$\delta_{t|t-1} = \delta_t - \delta_{t-1} \frac{(1 - m_t)^2}{(1 - m_{t-1})^2} \tag{7}$$

In the reverse process, the diffusion starts from the conditional image $y \in \mathbb{R}^{H \times W \times C}$ by setting the input $x_T = y = f_k(I^{input})$, where f_k denotes bilinear interpolation with an upsampling factor k. Given x_t, the previous state x_{t-1} is predicted by

$$p_\theta(x_{t-1} \mid x_t, y) = \mathcal{N}\big(x_{t-1}; \mu_\theta(x_t, t, y), \tilde{\delta}_t I\big) \tag{8}$$

where $\mu_\theta(x_t, t, y)$ is the predicted mean and $\tilde{\delta}_t$ is the variance at each diffusion step, which does not need to be learned, allowing the model to use a fixed variance schedule during sampling.

The training objective for the BBDM process is based on optimizing an Evidence Lower Bound (ELBO)[2], which can be written as:

$$
\begin{aligned}
ELBO = -\mathbb{E}_q\Big[& D_{KL}\big(q(x_T \mid x_0, y) \| p(x_T \mid y)\big) \\
& + \sum_{t=2}^{T} D_{KL}\left(q(x_{t-1} \mid x_t, x_0, y) \| p_\theta(x_{t-1} \mid x_t, y)\right) \\
& - \log p_\theta(x_0 \mid x_1, y)\Big]
\end{aligned} \tag{9}
$$

The term $q(x_{t-1} \mid x_t, x_0, y)$ in the second Kullback–Leibler (KL)[3] divergence can be derived using Bayes' rule and the Markov chain property:

$$
q(x_{t-1} \mid x_t, x_0, y) = \frac{q(x_t \mid x_{t-1}, y) q(x_{t-1} \mid x_0, y)}{q(x_t \mid x_0, y)} \tag{10}
$$

Using Eq. (1) and Eq. (6), the closed-form expression becomes

$$
q(x_{t-1} \mid x_t, x_0, y) = \mathcal{N}\left(x_{t-1}; \tilde{\mu}_t(x_t, x_0, y), \tilde{\delta}_t I\right) \tag{11}
$$

where the mean and variance are given by

$$
\begin{aligned}
\tilde{\mu}_t(x_t, x_0, y) \ = & \frac{\delta_{t-1}}{\delta_t} \frac{1 - m_t}{1 - m_{t-1}} x_t + \left((1 - m_{t-1}) \frac{\delta_{t|t-1}}{\delta_t}\right) x_0 \\
& + \left(m_{t-1} - \frac{m_t(1 - m_t)}{1 - m_{t-1}} \frac{\delta_{t-1}}{\delta_t}\right) y
\end{aligned} \tag{12}
$$

and

$$
\tilde{\delta}_t \ = \delta_{t|t-1} \frac{\delta_{t-1}}{\delta_t} \tag{13}
$$

Combining Eq. (3) with Eq. (12), the mean term can be reparametrized as:

$$
\tilde{\mu}_t(x_t, x_0, y) = c_x x_t + c_y y - c_\varepsilon\left(m_t(y - x_0) + \sqrt{\delta_t} \varepsilon_t\right) \tag{13}
$$

where

$$
c_x = \frac{\delta_{t-1}}{\delta_t} \frac{1 - m_t}{1 - m_{t-1}} + \frac{\delta_{t|t-1}}{\delta_t}(1 - m_{t-1}) \tag{14}
$$

$$
c_y = m_{t-1} - m_t \frac{1 - m_t}{1 - m_{t-1}} \frac{\delta_{t-1}}{\delta_t} \tag{15}
$$

$$
c_\varepsilon = (1 - m_{t-1}) \frac{\delta_{t|t-1}}{\delta_t} \tag{16}
$$

During training, the U-Net (i.e., ε_θ) learns to estimate the difference between the noisy sample x_t and the original x_0,

$$
x_t - x_0 = m_t(y - x_0) + \sqrt{\delta_t} \varepsilon_t \tag{17}
$$

Thus, we define

$$\tilde{\mu}_t(x_t, y) = c_x x_t + c_y y - c_\varepsilon \varepsilon_\theta(x_t, t) \tag{18}$$

Finally, the training loss is reduced to minimizing the squared error between the true forward noise and the predicted noise:

$$\mathcal{L} = \sum_{t=1}^{T} \gamma_t \mathbb{E}_{(x_0,y),\varepsilon_t}\left[\left\| m_t(y - x_0) + \sqrt{\delta_t}\varepsilon_t - \varepsilon_\theta(x_t, t)\right\|_2^2\right] \tag{19}$$

where γ_t is a weighting coefficient for each t. In this framework, we treat each timestep t with equal importance, and thus we set γ_t to 1.

During inference, we follow a hybrid stochastic-deterministic sampling strategy, combining the vanilla sampling and mean sampling approaches[4]. The vanilla sampling strategy follows the reverse process defined in Eqs. (8-18). To reduce variance during the late denoising phase, we switch to noise-free mean[4] (i.e., deterministic) sampling at an exit time point $t = t_e$. Given a sampling step t, the previous step $t-1$ is predicted as:

$$p(x_{t-1} \mid x_t, y) = \begin{cases} \mathcal{N}\left(x_{t-1}; \tilde{\mu}_t(x_t, y), \tilde{\delta}_t I\right), & t_e < t \leq T \\ \mathcal{N}\left(x_{t-1}; \tilde{\mu}_t(x_t, y), 0\right), & t \leq t_e \end{cases} \tag{20}$$

For the results presented in this work, the number of sampling steps T is set to 1000 for both training and blind inference. The exit point was empirically set to $t_e = 50$.

References

1 Li, B., Xue, K., Liu, B. & Lai, Y.-K. in *2023 IEEE/CVF Conference on Computer Vision and Pattern Recognition (CVPR)* 1952-1961 (2023).

2 Kingma, D. & Welling, M. *Auto-Encoding Variational Bayes*. (2014).

3 Kullback, S. & Leibler, R. A. On Information and Sufficiency. *The Annals of Mathematical Statistics* **22**, 79-86 (1951). https://doi.org:10.1214/aoms/1177729694

4 Zhang, Y. *et al.* Pixel super-resolved virtual staining of label-free tissue using diffusion models. *Nature Communications* **16**, 5016 (2025). https://doi.org:10.1038/s41467-025-60387-z

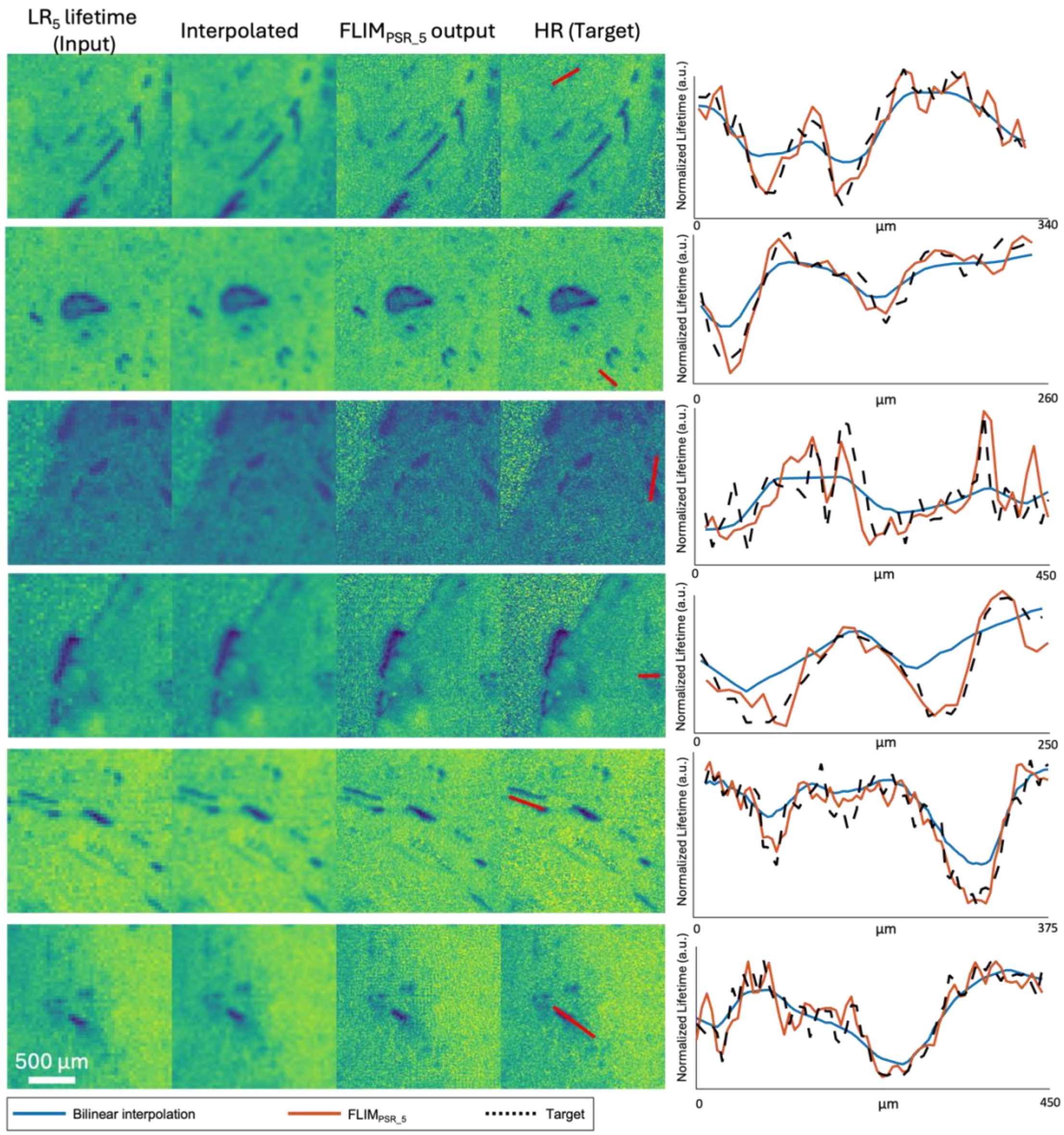

Figure S1. Additional Examples of the Structural Fidelity and Resolution Validation of $FLIM_{PSR\_5}$ Lifetime Images (also see Figure 2 of the main text). Representative lifetime channel examples at PSR factor k = 5. Low-resolution input (LR_5, **first column**), interpolated image (**second column**), $FLIM_{PSR\_5}$ output (**third column**), and HR target (**fourth column**). All images are shown with the same lifetime colormap and scaling. The shown fields of view correspond to channels LT_2, LT_1, LT_3, LT_1, LT_2, and LT_2, respectively. **(Right column)** Line profiles extracted along the paths indicated in the HR target regions. The $FLIM_{PSR\_5}$ output image profiles (solid red lines) closely follow the HR target traces (dashed black lines), recovering sharp lifetime transitions and local gradients, while the interpolated profiles (solid blue lines) remain smoothed and under-represent local variations.

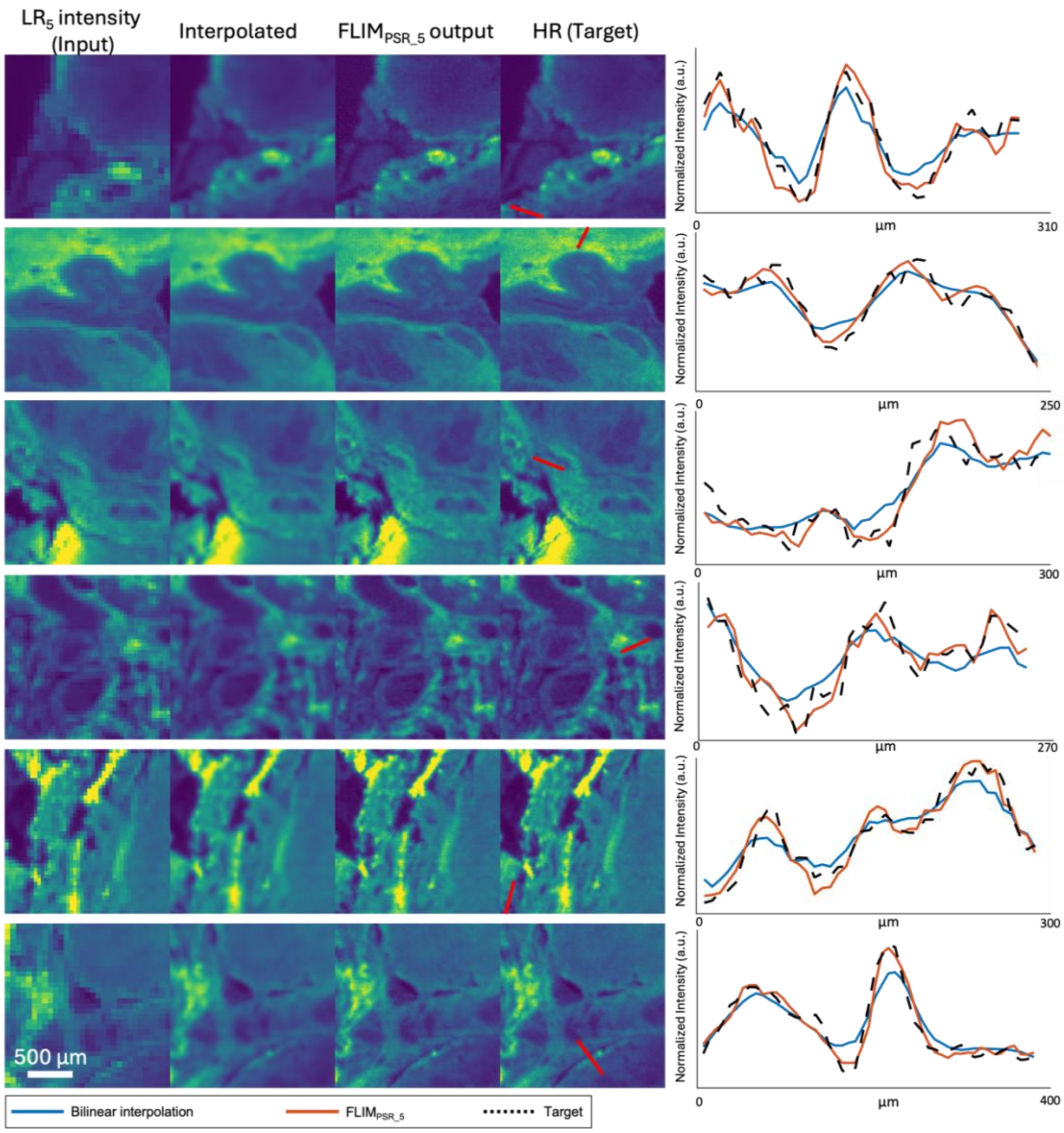

Figure S2. Additional Examples of the Structural Fidelity and Resolution Validation of $FLIM_{PSR_5}$Intensity Images (also see Figure 3 of the main text). Representative intensity channel examples at PSR factor k = 5. Low-resolution input (LR_5, **first column**), interpolated image (**second column**), $FLIM_{PSR\_5}$ output (**third column**), and HR target (**fourth column**). All images are shown with the same colormap and scaling. The shown fields of view correspond to channels INT_2, INT_1, INT_2, INT_1, INT_2, and INT_2, respectively. **(Right column)** Line profiles extracted along the paths indicated in the HR target regions. The $FLIM_{PSR\_5}$ output image profiles (solid red lines) closely follow the HR target traces (dashed black lines), recovering sharp intensity transitions and local gradients, while the interpolated profiles (solid blue lines) remain smoothed and under-represent local variations.

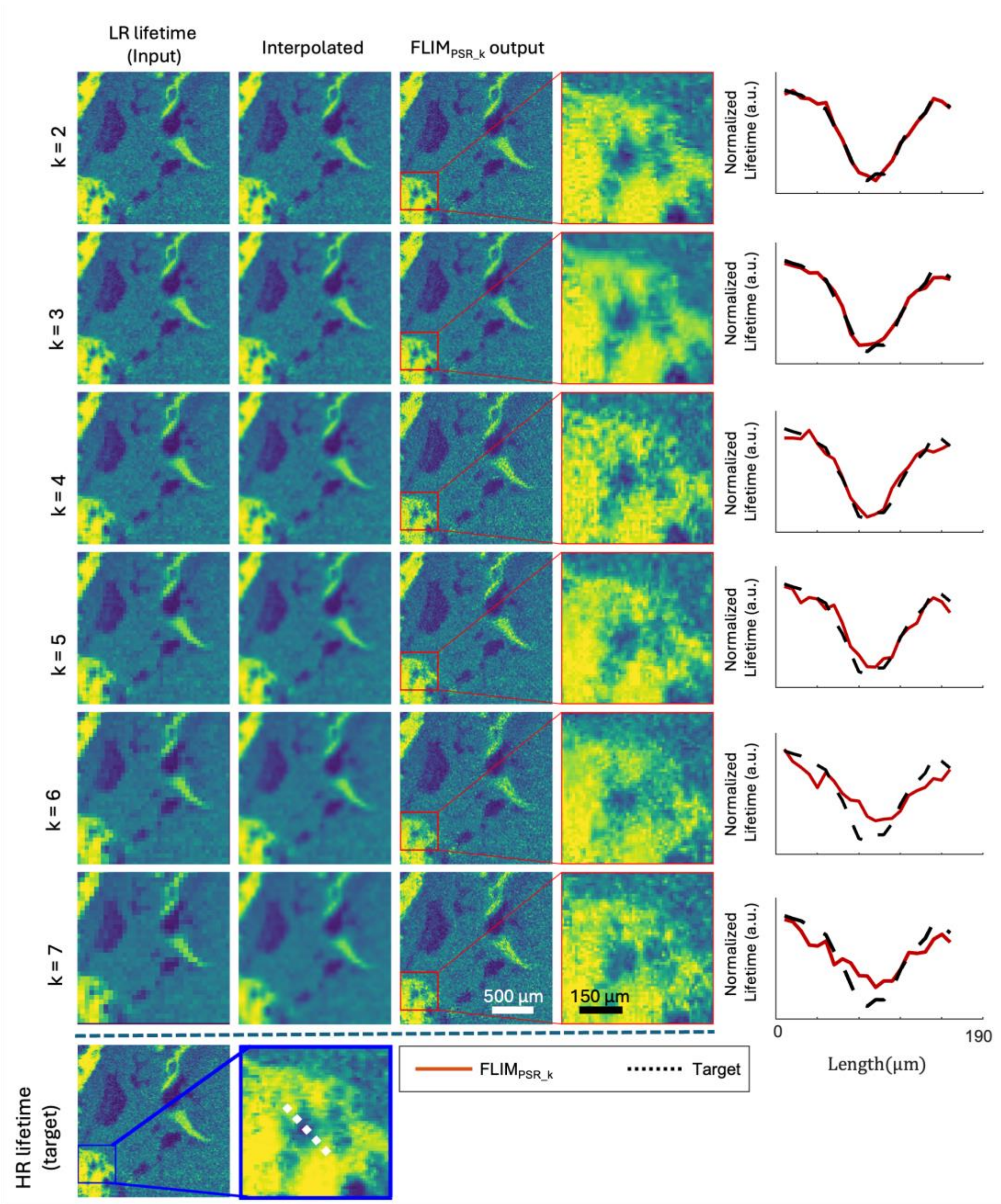

Figure S3. Additional Example of the Performance of Lifetime FLIM$_{PSR_k}$ Across PSR Factors (also see Figure 4 of the main text). Lifetime reconstructions for a representative field of view at PSR factors of k = 2,3,4,5,6 and 7. For each k, the panel shows the low-resolution input (LR), the interpolated image, and the FLIM$_{PSR_k}$ output, together with the HR lifetime target. Up to k=5, the PSR outputs retain clear tissue boundaries and local lifetime variations that match the HR reference FLIM image. Zoomed-in regions from the same field of view for all PSR factors are shown in the third column. Line profiles through the zoomed feature are shown for each PSR factor. The field of view shown corresponds to channel LT_3.

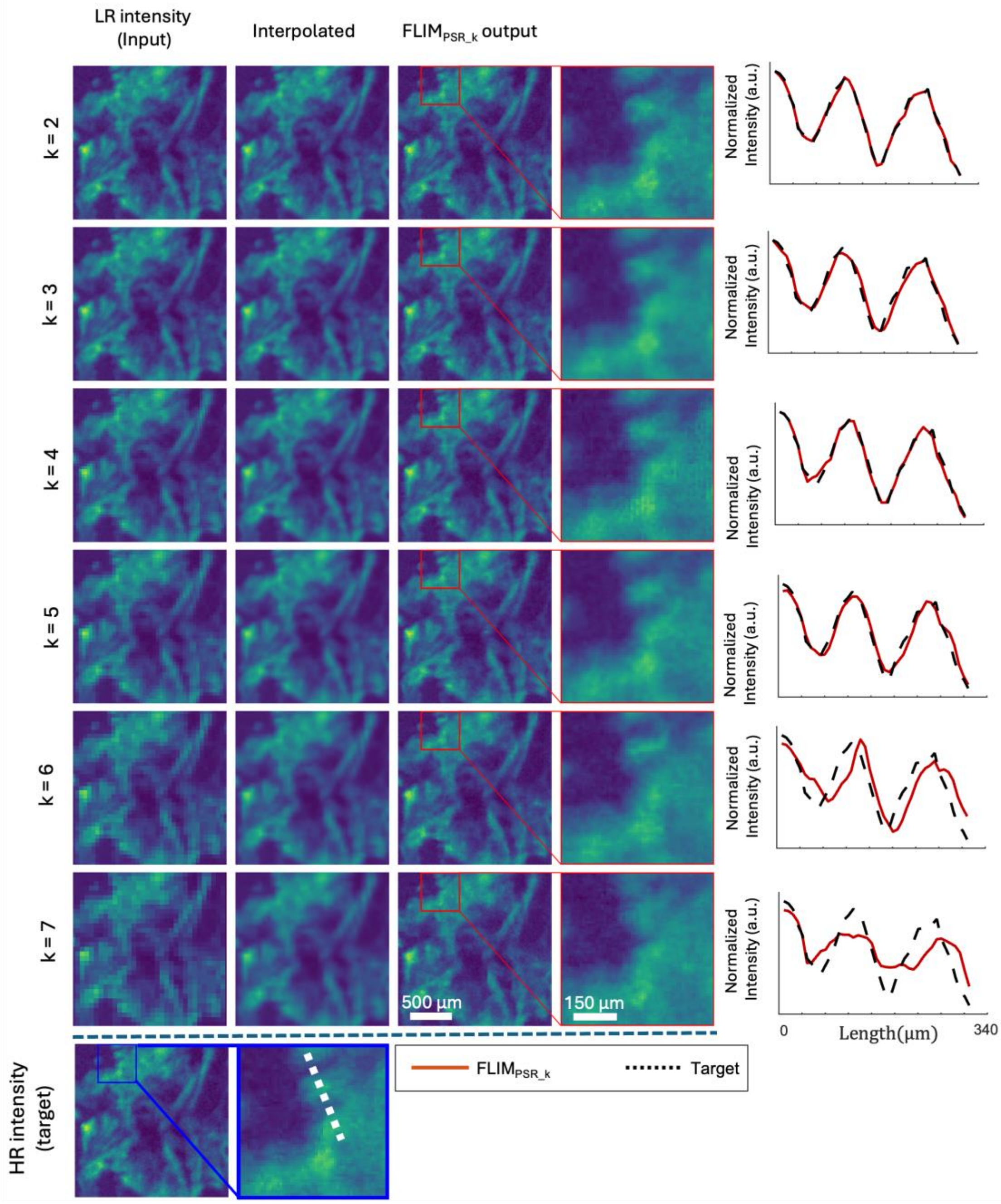

Figure S4. Additional Example of the Performance of Intensity FLIM$_{PSR_k}$ Across PSR Factors (also see Figure 5 of the main text). Intensity reconstructions for a representative field of view at PSR factors of k = 2,3,4,5,6 and 7. For each k, the panel shows the low-resolution input (LR), the interpolated image, and the FLIM$_{PSR_k}$ output, together with the HR intensity target. Up to k=5, the PSR outputs retain clear tissue boundaries and local intensity variations that match the HR reference FLIM image. Zoomed-in regions from the same field of view for all PSR factors are shown in the third column. Line profiles through the zoomed feature are shown for each PSR factor. The field of view shown corresponds to channel INT_1.

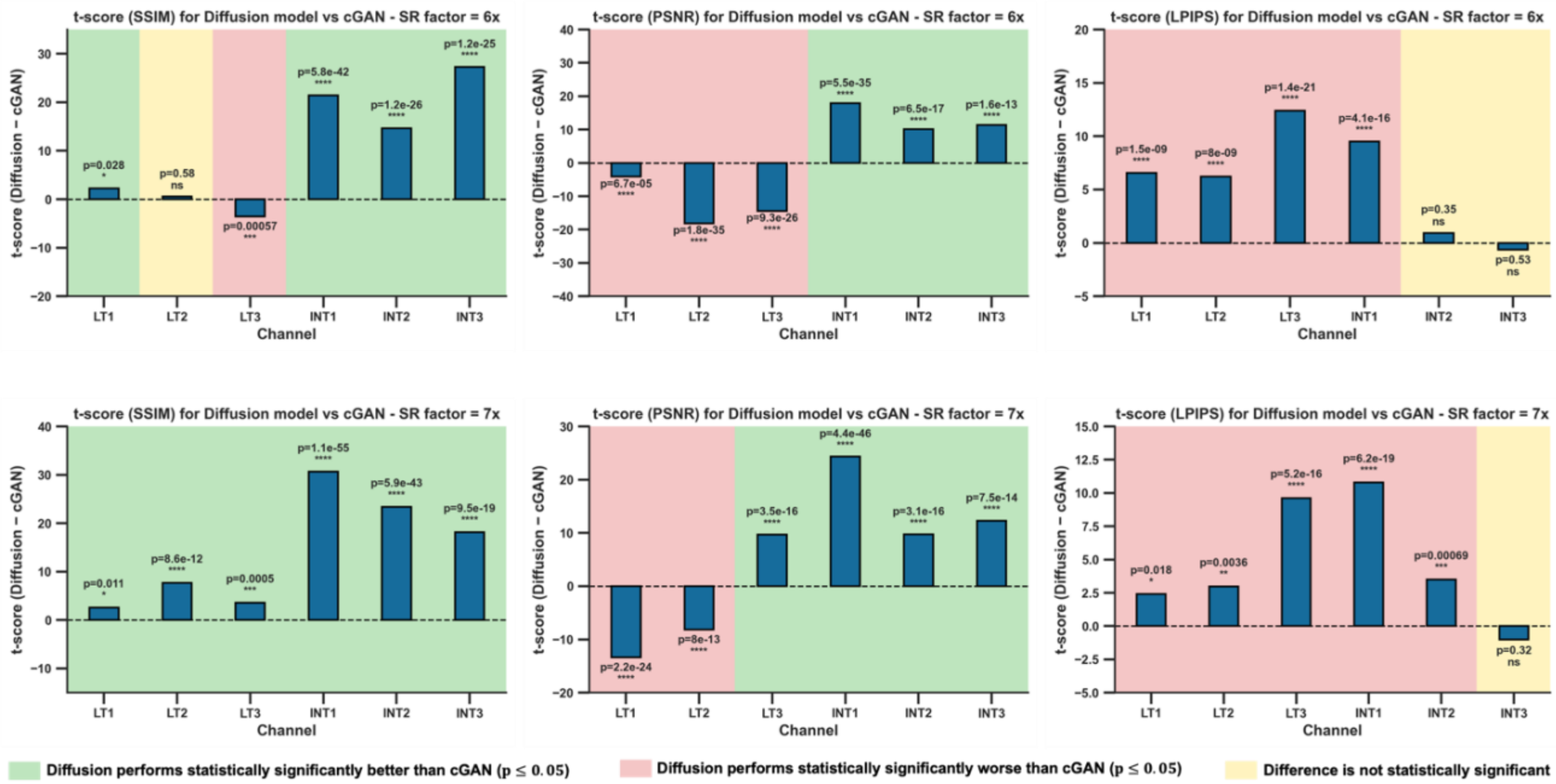

Figure S5. High PSR scale comparison of $FLIM_{PSR\_k}$ cGAN-based models and diffusion-based PSR models at k = 6 (top) and k = 7 (bottom). Paired two-sided t-tests were performed to evaluate whether diffusion models provided statistically significant differences in reconstruction quality relative to the cGAN framework for three image-quality metrics: SSIM (left column), PSNR (middle column), and LPIPS (right column). Each bar represents the t-score computed across 128 paired fields of view for a given FLIM channel. p-values are shown above each bar, with significance levels indicated by asterisks. Background shading highlights interpretation categories: green shading indicates statistically significant improvement for diffusion models over cGAN ($p \leq 0.05$, $t > 0$ for SSIM and PSNR, $t < 0$ for LPIPS), red indicates statistically significant improvement for cGAN over diffusion models ($p \leq 0.05$, $t < 0$ for SSIM and PSNR, $t > 0$ for LPIPS), and yellow indicates non-significant (ns) differences ($p > 0.05$).

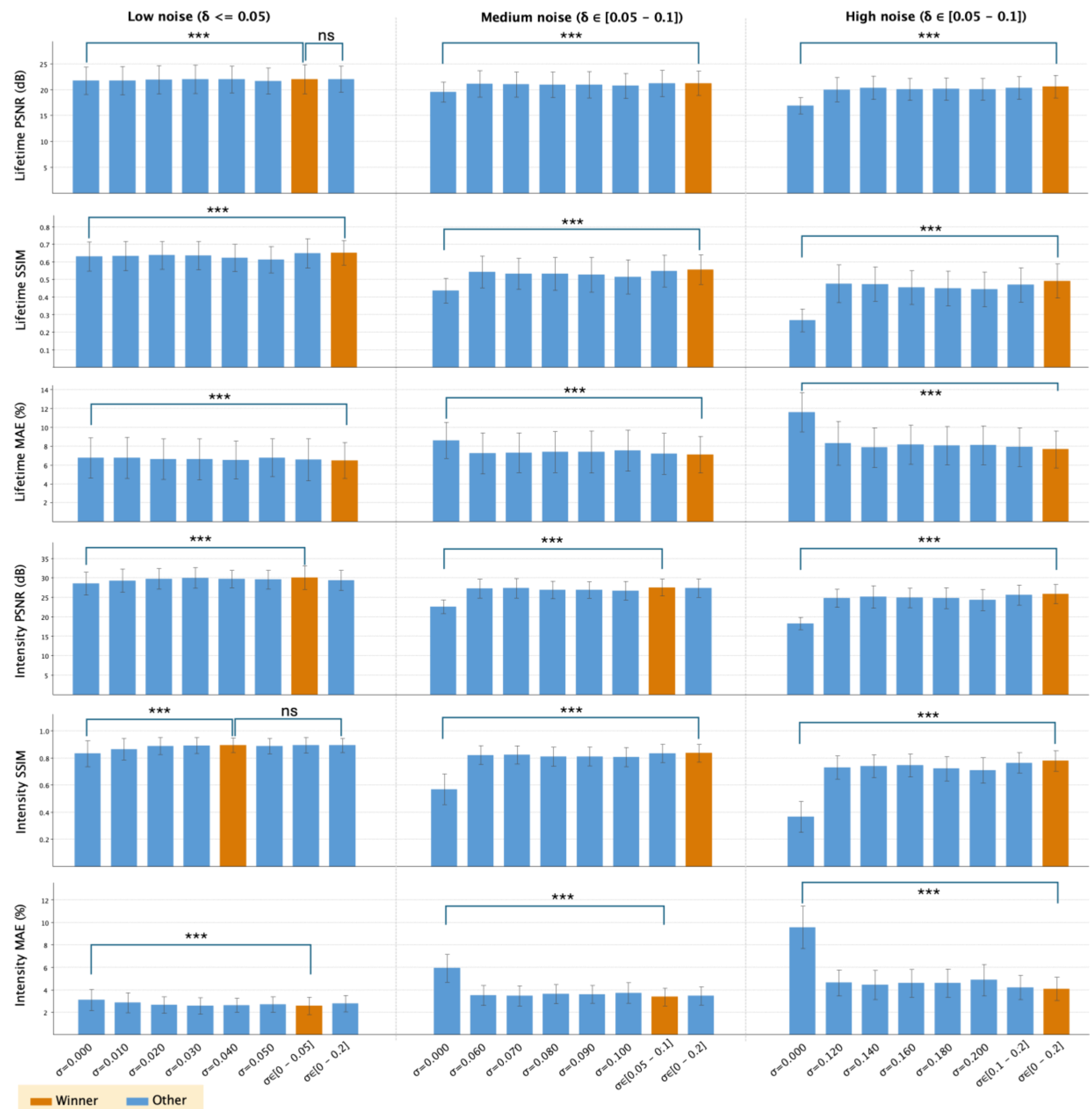

Figure S6. Quantitative comparison of noise vaccination strategies across noise levels and modalities. Bar plots show the reconstruction performance for $FLIM_{PSR\_5}$ models trained with different noise vaccination strategies, evaluated at low (δ ∈ [0, 0.05]), medium (δ ∈ [0.05, 0.1]), and high (δ ∈ [0.1, 0.2]) test noise levels (columns). Rows show PSNR, SSIM, and percentage mean absolute error (MAE) for lifetime channels (top three rows) and intensity channels (bottom three rows). Each bar represents a different model: baseline (no vaccination), models trained with fixed noise levels, models trained with narrow-range vaccination, and models trained with full-range vaccination (σ ∈ [0, 0.2]). Orange indicates the best-performing model for each condition based on the mean value of each performance metric. Every winner model is superior to the baseline model (no vaccination) with a strong statistical significance ($p < 0.001$). Error bars represent standard deviation across 128 test fields of view with 100 noise iterations per field

of view. The full-range vaccination model achieves high performance across all metrics and noise levels.

d

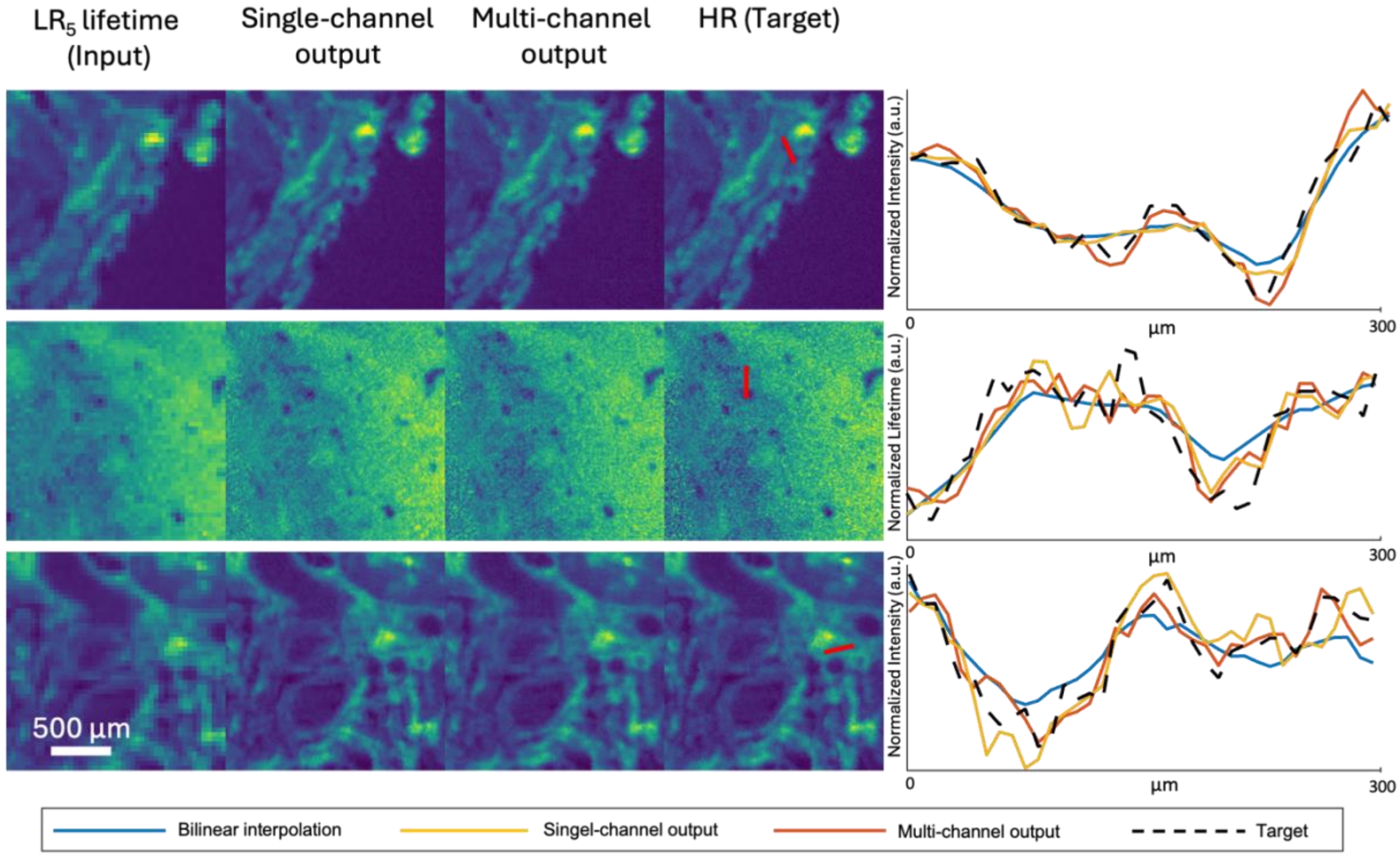

Figure S7. Comparison of multi-channel and single-channel FLIM pixel super-resolution models. Representative examples illustrating the differences between reconstructions produced by the multi-channel cGAN ($FLIM_{PSR\_5}$) and a single-channel model trained independently for FLIM channel 2. Three regions from head and neck tissue are shown (rows 1-3, INT_2, LT_2, and INT_2, respectively), each displaying the low-resolution input (k = 5), single-channel PSR output, multi-channel PSR output, and high-resolution target. Red markers indicate the location of the cross-section profiles plotted to the right. The line profiles compare the interpolated baseline (solid blue), single-channel output (solid yellow), multi-channel output (solid red), and high-resolution target (dashed black).
